# Antibody design using deep learning: from sequence and structure design to affinity maturation

**DOI:** 10.1093/bib/bbae307

**Published:** 2024-07-03

**Authors:** Sara Joubbi, Alessio Micheli, Paolo Milazzo, Giuseppe Maccari, Giorgio Ciano, Dario Cardamone, Duccio Medini

**Affiliations:** Department of Computer Science, University of Pisa, Largo B. Pontecorvo, 3, 56127, Pisa, Italy; Data Science for Health (DaScH) Lab, Fondazione Toscana Life Sciences, Via Fiorentina, 1, 53100, Siena, Italy; Department of Computer Science, University of Pisa, Largo B. Pontecorvo, 3, 56127, Pisa, Italy; Department of Computer Science, University of Pisa, Largo B. Pontecorvo, 3, 56127, Pisa, Italy; Data Science for Health (DaScH) Lab, Fondazione Toscana Life Sciences, Via Fiorentina, 1, 53100, Siena, Italy; Data Science for Health (DaScH) Lab, Fondazione Toscana Life Sciences, Via Fiorentina, 1, 53100, Siena, Italy; Data Science for Health (DaScH) Lab, Fondazione Toscana Life Sciences, Via Fiorentina, 1, 53100, Siena, Italy; Data Science for Health (DaScH) Lab, Fondazione Toscana Life Sciences, Via Fiorentina, 1, 53100, Siena, Italy

**Keywords:** antibody, nanobody, deep learning, antibody design, antibody optimization

## Abstract

Deep learning has achieved impressive results in various fields such as computer vision and natural language processing, making it a powerful tool in biology. Its applications now encompass cellular image classification, genomic studies and drug discovery. While drug development traditionally focused deep learning applications on small molecules, recent innovations have incorporated it in the discovery and development of biological molecules, particularly antibodies. Researchers have devised novel techniques to streamline antibody development, combining *in vitro* and *in silico* methods. In particular, computational power expedites lead candidate generation, scaling and potential antibody development against complex antigens. This survey highlights significant advancements in protein design and optimization, specifically focusing on antibodies. This includes various aspects such as design, folding, antibody–antigen interactions docking and affinity maturation.

## Introduction

Antibodies, versatile immune system proteins, have a remarkable ability to recognize foreign molecules (antigens) during adaptive immune responses [1]. Monoclonal antibodies (mAbs) have become a leading class of biotherapeutics due to their exceptional binding properties. In contrast to traditional small-molecule drugs, known for binding to multiple targets and causing off-target effects, mAbs exhibit high specificity and can be engineered to target specific disease-causing molecules. The realm of antibody-based therapeutics is witnessing rapid growth, with over 100 FDA authorizations achieved [2] and over 1000 in clinical studies [3]. This dynamic landscape has contributed to the remarkable market size. The antibody therapy market is projected to surpass 400 billion by 2028, with a compound annual growth rate of 14.1% [4].

Antibodies are usually developed using labor-intensive and expensive techniques [5–7]. To address these limitations, researchers have created computational methods to combine with standard *in vitro* approaches. However, these methods rely on sampling and scoring techniques [8] that have their drawbacks. These limits include dependency on databases with known structures, elevated computational costs and reliance on energy functions [9].

In response to these challenges, state-of-the-art methods based on deep learning (DL) [10, 11] have emerged. DL uses artificial neural networks with multiple layers to automatically learn hierarchical representations of data, enabling the development of highly flexible and powerful models for various tasks. One class of DL models is graph neural networks (GNNs) [12–14]—in this survey, we consider also graph convolutional network (GCN) as part of GNN—which extend traditional neural network architectures to handle graph-structured data. Another class of DL models considered in this survey is Transformers [15]. Transformers are known for their ability to capture long-range dependencies in all types of data, from sequences to images, using their attention mechanism. DL, which has achieved notable success in fields like computer vision (CV) and natural language processing (NLP), has firmly established itself as a potent methodology applicable to biology. In particular, DL models have the potential to accurately predict antibody structures, antibody–antigen (Ab–Ag) interactions and contribute to antibody generation. The advent of next-generation sequencing (NGS) technologies has also facilitated the comprehensive characterization of antibody repertoires, leading to the establishment of publicly accessible repositories for sequencing data [16] that can be used to train DL models.

This survey thoroughly investigates the major aspects of antibody design, starting with an introduction to antibodies and essential databases. It then explores the historical evolution of antibody-related methods, transitioning from traditional techniques to computational approaches, with a focus on associated limitations. Finally, the survey examines current state-of-the-art DL methods for different stages of *in silico* antibody development, including sequence/structure design, folding, paratope–epitope prediction, Ab–Ag docking and affinity  maturation and prediction. Finally, a brief discussion will be provided on DL and developability. However, the foundational principles of DL (and GNN) are not explicitly explored in this survey. Readers are encouraged to refer to key works such as [10, 11, 13, 14, 17, 18] for a more comprehensive exploration.

## The architecture of antibodies: key components and structural challenges

### Antibody generation and structure

**Figure 1:**
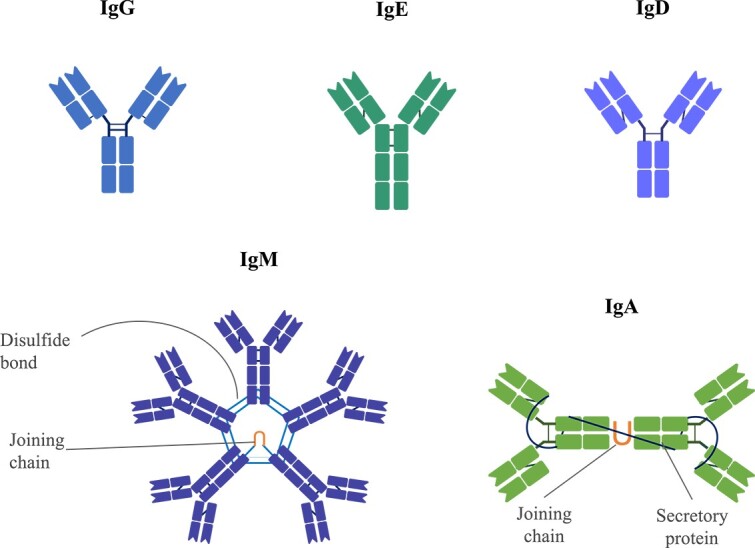
Illustration of distinct immunoglobulin structures: IgG (gamma), IgE (epsilon), IgD (delta), IgM (mu) and IgA (alpha).

Antibodies, also known as Immunoglobulins (Igs), are essential proteins produced in response to invading pathogens [19]. B-cells, which are a type of lymphocyte [20], generate antibodies through somatic recombination involving Variable (V), Diversity (D), Joining (J) and Constant (C) gene segments, resulting in an estimated diversity of around $10^{13}$ unique sequences [21]. This process yields Heavy (H) and Light (L) chains that form various structurally different antibody subtypes [19], such as IgG, IgE, IgD, IgM and IgA, as shown in Figure 1. Among these, IgG is the most prevalent circulating antibody in blood and extracellular fluid.

**Figure 2:**
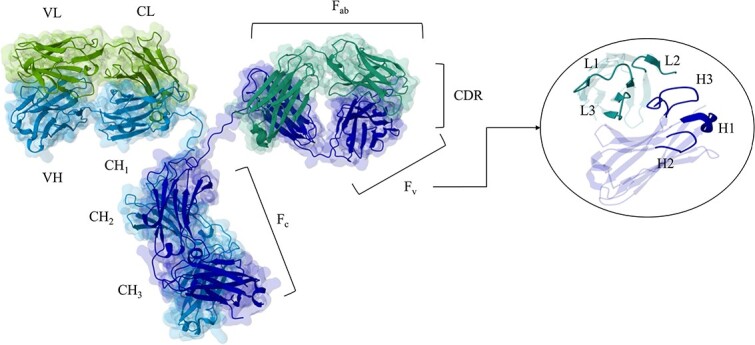
Ribbon diagram of an antibody structure (PDB 1IGT), with a focus on the variable region (PDB 1DQL). The heavy chain (H) of the antibody is depicted in light blue and dark blue, while the light chain (L) is shown in light green and dark green. On the right, a focus on a CDR is shown with labeled light and heavy chain CDR loops.

The structure of an Ig, as shown in Figure 2, includes a crystallizable fragment (F$_{c}$) that contains constant regions of the heavy chain (CH$_{2}$ and CH$_{3}$) and two antigen-binding fragments (F$_{ab}$). Within the F$_{ab}$ region, there are segments of the constant part of the heavy chain (CH$_{1}$) and the light chain (CL), along with a variable fragment (F$_{v}$). The F$_{v}$ includes the variable regions of the heavy (VH) and light (VL) chains. Both VH and VL chains contain three hypervariable loops, collectively known as complementarity-determining regions (CDRs).

### Antibody–Antigen interactions

**Figure 3:**
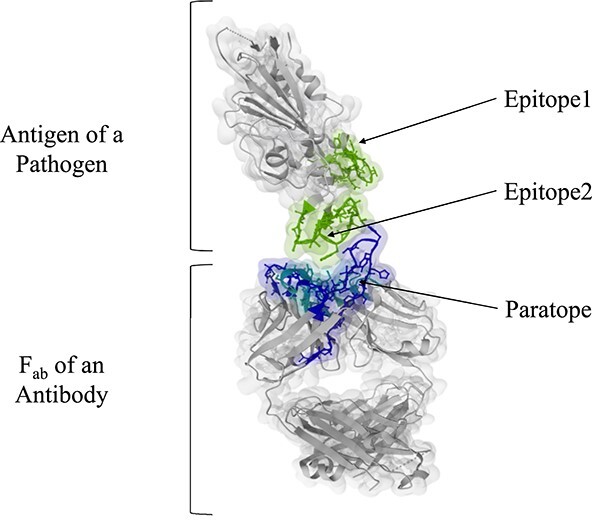
Paratope and epitope interaction. The pathogen’s surface can have multiple targets, each consisting of different antigens. Moreover, each antigen’s surface may exhibit multiple epitopes, which are the binding regions for the antibody’s paratope.

The variable domains (F$_{v}$) play a pivotal role as they constitute the antibody’s binding surface to the target antigen, known as the ‘paratope’ and ‘epitope’, respectively [22]—see Figure 3. The paratope is primarily composed of six distinct variable loops: L1, L2 and L3 on the light chain, and H1, H2 and H3 on the heavy chain as shown in Figure 2. These loops provide ample space for multiple unique contacts, contributing to the exceptional specificity of antibodies compared with small molecules [23, 24]. An epitope represents a specific site on the surface of a target, such as a protein, pathogen or cell. It serves as a recognition site for the immune system.

### Alternative formats of antibodies: nanobodies

In recent years, alternative antibody fragments have emerged as viable options to conventional antibodies (https://www.blopig.com/blog/2021/07/a-to-z-of-alternative-antibody-formats-next-generation-therapeutics/). For instance, single-chain variable fragments (scFvs), which contain both VH and VL, and nanobodies (VHHs), containing a single heavy chain. While scFVs are commonly used in clinical settings, VHHs have been shown to possess superior properties, as demonstrated in [25]. Indeed, this type of antibody has demonstrated high specificity, solubility, stability and low toxicity and immunogenicity [26–28]. They efficiently form high-affinity antigen complexes and hold promise for disease treatment and molecular imaging, including cancer and drug development [29–33]. While our main focus in this survey is on mAbs, we also discuss the rapidly expanding field of DL methods applied to nanobodies, which has experienced significant growth in recent years [34].

### Challenges in antibody development

Antibody development presents various challenges, starting with the design and structure prediction of the CDR H3 loop. The H3 loop undergoes independent mutation through V(D)J recombination before joining the rest of the antibody sequence [23], introducing variability and significantly affecting the structure and function of the antibody. Consequently, accurately predicting and modeling the H3 loop becomes more difficult.

Another intricate aspect is affinity maturation, a crucial process in antibody development. Antibodies undergo significant mutations that enhance their binding strength and specificity to target antigens compared with naive B-cell receptors [35]. Recreating this procedure is challenging as significant antibody mutations do not necessarily ensure improved binding strength and specificity.

## Databases for antibody development

DL requires extensive high-quality datasets to achieve optimal performance. Therefore, the creation of databases becomes crucial for training these methods. Consequently, multiple public databases of Ab sequences, structures and properties (e.g. binding affinity) have emerged, as listed in Table 1. These databases are regularly maintained and offer convenient access to raw data in the form of CSV files or PDB files. Some databases also provide analysis tools, such as abYsis [36].

**Table 1:** Existing public databases with antibody sequences, structures and properties (data from May 2024).

**Name**	**Data**	**Description**	**Ref**
AbDb	Sequence Structure	Extract Fv regions from antibody structures using the SACS database, which provides a summary of antibody PDB structures.	[37]
AB-Bind	Structure	Includes 1101 mutants with experimentally determined $\Delta \Delta $G changes in 32 complexes.	[38]
AbDiver	Sequences	Collects data from 900M immunoglobulin sequences (81 studies) for diversity exploration via positional profiling, V-region searches and clonotype examination.	[39]
abYsis	Sequence Structure	Bioinformatic tool perform various operations, including antibody sequence management, 3D structure integration, automated antibody numbering, canonical class annotation, unusual residue identification, humanization, germline view and user input.	[36]
Cov-AbDab	Sequence	Catalogs 12 916 entries of published/patented antibodies and nanobodies that bind to coronaviruses, such as SARS-CoV2 and SARS-CoV1.	[40]
INDI	Sequence Structure	Integrates nanobody sequences, structures and the metadata associated with them from all the major data repositories in the public domain (e.g. PDB, patent and scientific publications).	[41]
NanoLAS	Sequence Structure	User-friendly nanobody information database that provides extensive and diverse data sources. It also offers intuitive 3D visualization of nanobody structures and a comparative analysis feature, making scientific research and understanding easier.	[42]
OAS	Sequence	Houses over 2 billion immune repertoires from 90 studies, including diverse immune states, organisms and individuals, with unpaired and paired antibody sequences.	[43]
PAD	Sequence	Contains around 267 722 antibody chains sourced from primary patent documents and third-party patent documents.	[44]
PLAbDab	Sequence	Contains over 150 000 paired antibody sequences and 3D structural models from patent and academic papers.	[45]
SAbDab	Structure	Houses annotated antibody structures from the PDB, offering a consistent presentation alongside experimental details, affinity data and sequence annotations.	[46]
sdAb-DB	Sequences	Compiles single-domain antibody sequences from literature, online repositories (PDB and NCBI) and user contributions.	[47]
SKEMPI	Structure	Comprises thermodynamic and kinetic changes upon mutation in solved PPI complexes in the PDB.	[48, 49]
Thera-SAbDab	Sequence Structure	Includes World Health Organization-recognized antibody and nanobody therapeutics, matching structures in SAbDab with nearly identical sequences or identical variable domain sequences.	[50]

### Sequence databases

The latest advancements in NGS technologies have facilitated the comprehensive profiling of antibody repertoires. As a result, publicly available repositories have been developed including the observed antibody space (OAS) [43], which encompasses more than 2 billion sequences. Additional information can be found in Table 1.

Moreover, various tools aid sequence analysis and exploration. PAD [44] gathers unpaired antibody sequences from patents, while PLAbDab [45] offers over 150 000 paired antibody sequences from patents and academic works.

### Structure databases

Understanding the structural information of antibodies is extremely important in the field of antibody design, offering crucial insights into their functionality and efficiency. The Protein Data Bank (PDB) is a crucial protein structural database that contains over 200 000 experimentally validated structures, including approximately 10 000 antibody structures. Various datasets are curated to extract antibody structures from the PDB, adding additional information. One such example is the Structural Antibody database (SAbDab) [46], which encompasses all publicly available antibody structures presented and consistently annotated. Each structure in SAbDab is enriched with various annotations, including experimental details, antibody nomenclature (such as heavy-light pairings), curated affinity data and sequence annotations. Additional resources, such as the Antibody Structure Database (AbDb) [37], abYsis [36] and the Therapeutic Structural Antibody Database (Thera-SAbDab) [50], are listed in Table 1.

Various specialized datasets are available for exploring antibody properties. For instance, the Structural Database of Kinetics and Energetics of Mutant Protein Interactions (SKEMPI) [48] can be used to investigate mutations’ binding free energy. An updated version, SKEMPI v2 [49], has been introduced, which provides meticulously verified binding information for 7085 mutations. Additionally, there are targeted repositories designed to meet specific research requirements, such as CoV-AbDab [40], which is exclusively dedicated to anticoronavirus antibodies.

Lastly, several nanobody databases, such as INDI [41] and NanoLAS [42], have been created to store all the information related to this specific subclass of antibodies.

## History of antibody development techniques

Before the rise of DL-driven methods, antibody design and development relied on wet-laboratory technologies and classical computational methods.

### Traditional techniques for antibody development

In the field of antibody development, conventional experimental approaches have long served as the foundation for the discovery and engineering of therapeutic antibodies. These methods involve techniques such as immunization and directed evolution through phage or yeast display [19, 23, 24]. Specifically, in the realm of vaccine development, vaccines and antibodies were traditionally developed by isolating and inactivating disease-causing microorganisms or their components [51]. The advent of genome sequencing enabled the discovery of new antigens directly from genomic information, leading to the concept of reverse vaccinology [51]. Recently, advancements in human immunology and structural biology have led to a new approach known as reverse vaccinology 2.0, enabling high-throughput screening of antibody-secreting cells (ASCs) to obtain neutralizing antibodies (nAbs) for prophylaxis or treatment [51].

#### Traditional techniques’ limitations

Traditional techniques for antibody development, although successful in generating antibody binders, have several limitations:

1. *ASC Selection*: The effectiveness of ASCs cloning process is limited by the diverse immune responses of individuals, capturing only a fraction of potential paratopes for the same epitope. Therefore, acquiring ASCs from thousands of subjects is necessary to achieve a comprehensive spectrum. However, this process is expensive and time-consuming;
2. *Selection of Specific Epitopes*: The selection process lacks control over the specific target molecule for which antibodies are chosen. If dominated by ‘strong’ epitopes, it may exclude other desirable targets. For example, less dominant epitopes on a highly conserved protein could be ideal targets;
3. *Problem of Target Variety*: Developing neutralizing mAbs becomes exceptionally challenging in situations with a high number of potential targets, such as bacteria, which have hundreds of different antigens;
4. *Time-consuming Optimization*: The selected antibodies may require further research and development to enhance their potency. This could include epitope mapping to study Ab–Ag interactions, which requires techniques such as X-ray crystallography or Nuclear Magnetic Resonance. However, these techniques are time-consuming and low-throughput.

### Pre-DL computational approaches for antibody development

To overcome previous limitations, computational methods for antibody design have emerged. *In silico* techniques play a pivotal role throughout the antibody discovery process, ranging from *de novo* design to developability assessment. Traditionally, antibody structures have been predicted using mechanistic modeling techniques such as molecular dynamics (MD) simulations [52], homology-based modeling [53] or a combination of these methods such as MODELLER [54]. An example of a conventional structure-guided antibody design, known as RosettaAntibodyDesign (RAbD) [8], uses alternating outer and inner Monte Carlo cycles. In each outer cycle, a CDR is randomly selected for design. The inner cycle consists of N rounds of sequence design, structural optimization and optional docking to enhance interactions with antigens. After each inner cycle, the new sequence and structure are accepted based on the Metropolis Monte Carlo criterion. This process is repeated for N rounds, with the resulting design’s energy compared with the previous one in the outer cycle. A homology-based model for structure prediction called ABodyBuilder [53] is composed of four key steps: template selection, VH-VL orientation prediction, CDR loop prediction and side-chain prediction. The above-mentioned methods represent a non-exhaustive overview of pre-DL computational approaches for antibody development. It is essential to acknowledge the existence of other significant and valid methods, such as AbDesign[55]. An overview of these methods can be found here [19].

#### Pre-DL methods limitations

Despite the advantages of using the described computational methods to support traditional techniques, they have the following limitations:

1. *Limited Focus on Variable Domain Sequence*: Most computational techniques focus on the antibody variable domain sequence, lacking structural data and limiting accuracy in predicting antibody structures;
2. *Focus on Heavy Chain*: Many sequencing experiments focus only on the heavy chain while overlooking the valuable information of the light chain, which is valuable for a comprehensive understanding of antibody development;
3. *Time-Consuming Process*: Mechanistic simulations struggle due to their time-consuming nature in accurately representing biomolecular processes. For instance, simulating 1 ms of dynamics in systems comprising about 50 000 atoms demands several days using a single GPU [56]. This atom count is significantly lower than the number involved in the paratope–epitope complex, estimated to be around 300 000 atoms (CR3022 antibody and SARS-CoV-2 RBD spike protein) [57];
4. *Problem in Using Structures*: The mentioned techniques depend on antibody structures, but their scarcity compared with sequences poses a challenge since traditional methods like X-ray are time-consuming and costly.

## DL for protein and antibody design

DL has a long history in the Neural Network field [58, 59] and has recently shown remarkable success in areas such as CV and NLP. This technique has extended its influence into the field of biology [60], paving the way for significant advancements in cellular image analysis [61–63], genomic studies [64–66],and drug discovery [67–70]. DL has found applications in antibody engineering, a critical aspect of therapeutic drug development [71]. By integrating DL methodologies with traditional experimental workflows, researchers aim to overcome *in vitro* and *in silico* limitations previously discussed. DL methods offer promise for more effective and scalable antibody-based biotherapeutics by accurately predicting antibody structures, Ab–Ag interactions and generating lead candidates. This section presents the state-of-the-art DL methods in antibody design.

**Figure 4:**
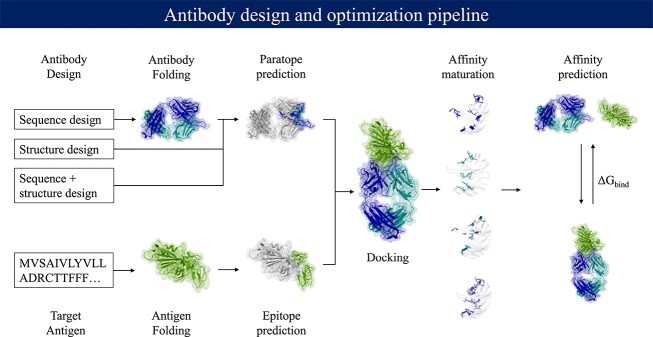
Overview of an *in silico* structure-based antibody design process. The antibody and antigen structures are taken from the PDB file 7T72.

Figure 4 shows the design and optimization process of an antibody. The first step is antibody design, which can be accomplished through either generating Ab sequences or structures. Then, the antibody–antigen complex undergoes modeling, which may involve separate steps for the antibody and antigen structures, paratope (Ab)-epitope (Ag) prediction and Ab–Ag docking. The resulting antibody constructs are evaluated based on affinity maturation and binding affinity. This iterative process continues until a suitable antibody construct is achieved. Finally, evaluating the developability of the designed structures and sequences can ultimately reduce the cost, time and effort required for experimental evaluation and successful commercialization.

In each subsection, different methods will be presented along with a qualitative and quantitative evaluation. The reader needs to note that the test and benchmark datasets used may differ among these methods. Consequently, these results should be viewed as indicative rather than used to compare each method directly. This applies unless otherwise specified in the caption of the figures and tables.

### Revolutionizing antibody design: the confluence of DL with structural and sequence information

**Figure 5:**
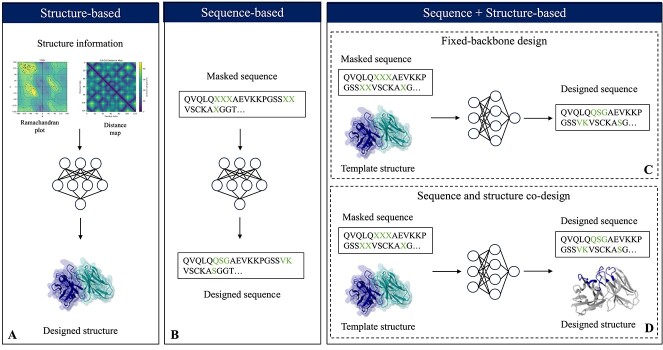
DL-based antibody generation falls into three categories. (A) Structure-based methods can create antibody structures, often beginning with structural information like contact maps. (B) Sequence-based methods generate antibody sequences, often by initiating with a masked sequence. Sequence + structure methods have two subcategories: (C) fixed-backbone, where the model mutates the input sequence to fold like a template (inverse-folding problem), and (D) co-design, where both sequence and structure are mutated. The antibody structure is from the PDB file 1DQL.

In the field of antibody design and protein engineering, the use of DL techniques has introduced new approaches that bridge the gap between structure and sequence information. These methods use DL to generate and manipulate both the structural and sequence aspects of antibodies, opening up new paths for tailored antibody development. Figure 5 displays three categories of DL models for Ab generation: structure-based, sequence-based and sequence + structure-based.

#### Structure-based DL models

Antibody design using structures, which focuses on the CDRH3 loops due to their variability, employs two distinct methodologies as outlined in [24]. The first method involves generating 3D coordinates to design realistic backbones for CDR-H3s [72], while the second predicts changes in $\Delta \Delta $G in the loops [73]. An example of the first category is Ig-VAE [72], which uses a Variational AutoEncoder (VAE) [74] to embed and reconstruct novel antibody backbone structures. This method can be constrained by specified structural elements. The process includes computing Ramachandran angles and distance matrices from full-atom backbone coordinates, passing them through an encoder–decoder network, and back-propagating errors to refine the generated structures (as shown in Figure 5A). Ig-VAE also achieved rotation and translation invariance using structure-derived information for backbone generation. An example of predicting changes in ΔΔG is provided by Shan *et al*. [73], who investigate changes in $\Delta \Delta $G in the binding affinity caused by amino acid substitutions. This specific method will be further discussed in the affinity maturation section. However, these models, which solely focus on the backbone, cannot incorporate specific epitopes and depend on external tools like Rosetta to predict mutational effects, as discussed in [24]. The comparison of these two methods is shown in Table 2.

**Table 2:** Comparison of antibody structure design models. The first model type uses structural components to compare the generated structures with the original one. In the second model, the authors employed the Pearson correlation coefficient (R) between the model-predicted $\Delta \Delta $G and the experimental $\Delta \Delta $G. (BL = bond length, BA = bond angle, SM = single mutation, MM = multiple mutations)

**Name**	**Class**	**Model**	Training Dataset	**Performance**	**Description**	**Ref**
lg-VAE	Antibody	VAE	AbDb/abYbank 10k sequences	$\phi\ \pm \sim $ 10$^{\circ }$ $\psi\ \pm \sim $10$^{\circ }$ $\omega \ \pm \sim $3$^{\circ }$ BL $\pm \sim $0.1Å BA $\pm \sim $10$^{\circ }$	**Strengths**: Generates 3D coordinates directly; rotational and translational invariance. **Limitations**: Depends on external tools. **Applications**: Antibody backbone generation.	[72]
Shan *et al*.	Antibody	Transformer	SKEMPI V2.0 5k mutations	R SM: 0.65 R MM: 0.59	**Strengths**: The attention network learns to identify key residue pairs near the protein interface that contribute to binding affinity. **Limitations**: Operates at the residue level and does not consider the atom level. **Applications**: Prediction of mutational effects on binding affinity.	[73]

#### Sequence-based DL models

Since obtaining antibody structures can be challenging, certain DL models are designed to capture extensive antibody features exclusively from their sequences (as shown in Table 3 and in Figure 1B).

**Table 3:** Models for antibody sequence design. In this context, these models cannot be compared with a single specific task. Therefore, we have compared them in Figure 6 based on the number of parameters, dimension of the embedding, and number of layers. In the following table, we compare the dimensions of the dataset, strengths, limitations, and applications. The ’Training Dataset’ column displays the number of sequences used for training.

**Name**	**Class**	**Model**	Training Dataset	**Description**	**Ref**
AbLang	Antibody	RoBERTa	OAS 14M	**Strengths**: Multiple representations (e.g. residue codings, sequence codings). **Limitations**: Lower performance in restoring longer regions and N-terminus residues. **Applications**: Restoring missing residues.	[88]
AntiBERTa	Antibody	RoBERTa	OAS 58M	**Strengths**: Captures critical aspects of BCRs like mutation count, V gene origin, and B cell type for specialized understanding. **Limitations**: Limited ability to predict paratopes in an antigen-specific manner due to data constraints. **Applications**: Paratope prediction and BCR repertoire analysis tasks.	[86]
AntiBERTy	Antibody	BERT	OAS 558M	**Strengths**: Reveals repertoire trajectories and efficiently detects redundant sequences and emphasizes critical binding residues. **Limitations**: Lacks the ability to differentiate between different species. **Applications**: Affinity maturation; Identifying key binding residues.	[85]
ESM-1b ESM-2	Protein	Transformer	UniRef50 250M	**Strengths**: Learning from large-scale data generalization and adaptability improved performance and scalability. **Limitations**: Dealing with a large model is challenging without sufficient resources. **Applications**: ESM-1b: prediction of mutational effect and secondary structure; ESM2: filling missing amino acid and structure prediction.	[82, 84]
IgLM	Antibody	Transformer	OAS 558M	**Strengths**: Produces infilled residue spans at designated positions within the antibody sequence. **Limitations**: Less effective at generating light chain sequences for most species. **Applications**: Designing sequences with improved developability and reduced immunogenic risks for various species or chain types.	[92]
nanoBERT	Nanobody	AntiBERTa	INDI 10M	**Strengths**: Demonstrated better performance for nanobodies compared with human-based methods. **Limitations**: Poor performance in distinguishing human sequences from non-human ones. **Applications**: Predicts the amino acid at a given position in a sequence.	[87]
Progen2-OAS	Antibody	Transformer	OAS 554M	**Strengths**: Model size comparable with protein language models. **Limitations**: Performs poorly compared with models pre-trained on protein databases. **Applications**: Ab sequence generation.	[89]
ProtBERT	Protein	BERT	UniRef100 216M	**Strengths**: Embeddings capture constraints relevant for protein structure and function. **Limitations**: BERT-based language models like AntiBERTa seem to perform better on Ab. **Applications**: Multiple protein tasks for per-residue or per-sequence predictions.	[80]

Protein sequences exhibit similarities to human languages, as noted in previous studies [75, 76]. This similarity has led to the development of NLP techniques tailored for encoding and using protein information [77, 78]. Among these techniques, Transformers have become the primary model for NLP due to their ability to capture long-range relationships in sequences [15]. Particularly, Transformer-based models like BERT have been instrumental in adapting NLP architectures for protein sequence analysis [79]. This adaptation led to the ProtBERT model [80], which was trained directly on datasets like Uniref100 [81]. ESM-1b [82] is a protein language model (pLM) trained on 86 billion amino acids from 250 million protein sequences through unsupervised learning. The obtained embeddings (see [83] for more details) encapsulate critical biological attributes, such as secondary and tertiary structure information. Expanding on ESM-1b, ESM-2 [84] encompasses a parameter range of 8 million to 15 billion, introducing improvements in architecture, training parameters, increased computational resources and expanded data compared with its predecessor. ESM-2 is notably utilized in ESMFold [84] for directly predicting protein structures from sequences.

**Figure 6:**
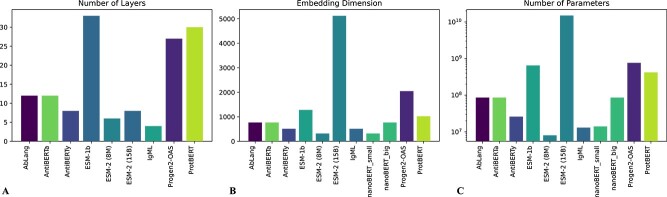
Models for designing antibody sequences. In this context, these models cannot be compared with a single specific task. We chose to compare them based on the number of layers (A), the embeddings dimension (B) and the number of learnable parameters (in logaritmic scale) (C). The dataset dimension is in Table 3.

A pLM trained specifically on antibody sequences is AntiBERTy [85], a BERT-based model trained on 558 million antibody sequences. This model’s embeddings cluster into directed evolution pathways and exhibit the capacity to identify paratope binding residues. AntiBERTa [86] is a masked language model comprising 86 million parameters. It was pre-trained on a dataset encompassing 67 million antibody sequences, including both heavy and light chains. The representations obtained from AntiBERTa were utilized for paratope prediction, demonstrating superior performance compared with ProtBERT, with Matthew’s correlation coefficient (MCC) values of 0.659 and 0.652, and an Area Under the ROC curve (ROC-AUC) values of 0.961 and 0.959, respectively. Following the trend of BERT models, Hadsund *et al*. [87] created nanoBERT, a nanobody-specific transformer for predicting amino acids at specific positions in a query sequence. NanoBERT outperforms human models by approximately 12% in V region reconstruction accuracy, with 76%, demonstrating the benefits of domain-specific language models. AbLang [88] is a pLM trained on OAS antibody sequences, which proves effective in filling missing residues in antibody sequence data, addressing a common issue in B-cell receptor repertoire sequencing. AbLang outperforms the general pLM ESM-1b in restoring missing residues, offering a faster alternative that does not rely on prior knowledge of the antibody germline. ProGen2-OAS [89], a fascinating outcome of the ProGen2 [90] lineage built upon the Generative Pretain Transformer-2 (GPT-2) [91] architecture, emerges as a transformer model trained on a vast dataset of 554 million antibody sequences. Lastly, IgLM [92] is a pLM based on GPT-2. It has been trained on 558 million antibody VH and VL sequences. IgLM is capable of generating complete antibody sequences across species and constructs infilled CDR loop libraries with improved *in silico* developability profiles. IgLM outperforms ProGen2-OAS and Progen2 (AUROC of 0.96 for IgLM, 0.94 for ProGen2-OAS and 0.87 for Progen2) in distinguishing between human and non-human antibodies, despite having significantly fewer parameters (13M for IgLM, 764M for ProGen2-OAS and 6.4B for ProGen2). These methods are compared in Figure 6. The strengths, limitations and applications of the methods presented in this section are shown in Table 3.

#### Structure- and sequence-based models

**Figure 7:**
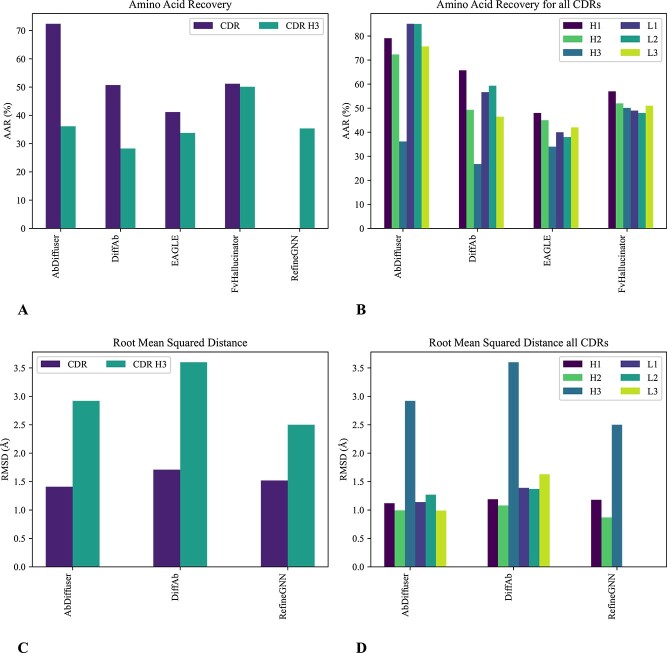
Comparison of CDR models for sequence and structure design, including diffusion and hallucination models. This comparison focuses on Amino Acid Recovery (AAR) for CDRs and CDRH3 (A), as well as all six CDRs (B), measuring the similarity between the actual and generated sequences. Additionally, Root Mean Squared Distance (RMSD) is used to assess the similarity between the real and generated structures, specifically for CDRs and CDRH3 (C), as well as all six CDRs (D). In cases where the CDR value is undefined, we considered the average metric value across all six CDRs. As expected for the majority of the methods, the AAR of CDRH3 is lower compared with the overall CDRs, while the RMSD is higher. This shows the challenges associated with designing the CDRH3 region. For a qualitative evaluation, refer to Table 4.

The fusion of structural and sequence information represents a promising frontier in DL-based antibody design (Table 4 and Figure 7). By bringing together these complementary sources of information, DL models can decipher intricate relationships between sequence variations and structural adaptations, providing insights into the complex interplay between form and function in antibodies. For instance, RefineGNN [93] is an autoregressive (AR)-based model for antibody generation that iteratively refines both the sequence and predicted global structure (sequence and structure co-design, Figure 5D). The inferred structure guides the selection of subsequent residues through a graph representation of amino acid positions and backbone structure angles, yet the existing model lacks consideration for specific epitopes. This model has been used for designing antibodies against SARS-CoV-1 and SARS-CoV-2.

**Table 4:** Comparison of sequence and structure design. The quantitative comparison of performance in CDR design, refer to Figure 7. In the ‘Training Dataset’ column the number of sequences (seq.) and structures (struct.) used for training is shown.

**Name**	**Class**	**Model**	Training Dataset	**Description**	**Ref**
AbDiffuser	Antibody	DDPM	pOAS (105k seq.) HER2 [104] (9k struct.)	**Strengths**: Can handle variable length sequences. **Limitations**: Does not consider the antigen or the epitope. **Applications**: Full Ab 3D structure and sequence design of variable length.	[101]
DiffAb	Antibody	DDPM	SAbDab	**Strengths**: Side-chains orientations design. **Limitations**: Relies on an Ab framework bound to the target Ag. **Applications**: Sequence-structure co-design, sequence design of CDRs for given backbone structures, and Ab optimization.	[100]
EAGLE	Antibody	DDPM	OAS (100M seq.) SAbDab (8k struct.)	**Strengths**: Use of sequence embedding and CLIP models with Ag structure. **Limitations**: CLIP has limited impact on model performance; requires knowledge of both the antigen and the epitope. **Applications**: Ab sequence designed conditioned on the Ag structure.	[102]
FvHallucinator	Antibody	DeepAb	AbDb abYbank (11k struct.)	**Strengths**: Designs substitutions highly enriched in human repertoire; integrated folding model. **Limitations**: Does not consider the Ag and optimization. **Applications**: Generate libraries of Ab sequences with fixed structure.	[96] [105]
RefineGNN	Antibody	GNN	SAbDab	**Strengths**: Modifies a generated subgraph to accommodate new residues. **Limitations**: Does not consider the epitope. **Applications**: Sequence and structure co-design of CDRs with enhanced binding specificity or neutralization capabilities.	[93]

In the realm of protein design, recent advancements in DL have shown promise, particularly in adapting successful techniques for antibody design tasks, such as hallucination [94] and diffusion models [95].

**Hallucination** Hallucination uses a pre-existing machine learning model to generate 3D protein structures from random sequences by predicting alpha-carbon distances and then refining the structures, followed by a refinement process through Monte Carlo simulations introducing mutations. The refinement process aims to make the generated structures more similar to authentic protein folds.

FvHallucinator [96] is a sequence design approach that extends the hallucination-based protein design to antibody-variable domain design, producing libraries of Fv sequences using a reference structure (fixed-backbone design, Figure 5C). The performance of this model drops significantly without wild-type seeding, to approximately 15–50% (H3 amino acid recovery). The limitations of hallucination techniques have become evident in experiments. Structures generated using these methods often fail to yield properly folded proteins in laboratory settings. These techniques also face difficulties in designing larger molecules, as they primarily focus on smaller proteins [97].

**Diffusion** Diffusion involves introducing noise to protein representations until they become Gaussian noise. Afterward, a DL model is trained to reverse this process and transform the noise into realistic protein structures. For protein design, one of the most interesting methods is RFdiffusion [98], which is built on a fine-tuned version of RosettaFold [99]. Recent developments in this field for Ab design include DiffAb [100], a deep generative model that combines Denoising Diffusion Probabilistic Model (DDPM) and equivariant neural networks for sequence and structure co-design of CDRs. However, DiffAb requires a starting structure of the antibody framework relative to the antigen. On the other hand, AbDiffuser [101] can independently co-design sequences and structures of variable length, eliminating the need for a starting structure. However, it does not consider the antigen or the epitope.

Cohen *et al*. [102] introduce EAGLE, a novel diffusion-based model for antibody sequence design. EAGLE can generate antibody sequences of various lengths using ESM embeddings, operating in a continuous space without requiring input backbone structures. The model incorporates epitope structure information during training through a CLIP module [103].

While diffusion models excel in shaping proteins with simple functions, they face challenges with complex structures like antibodies and struggle to create entirely novel designs [97].

### Advancements in antibody structure prediction and the role of DL

Following the development of a novel antibody sequence, a crucial next step is to define its structure. Understanding the complexities of antibody structures is essential for gaining knowledge of their specific characteristics, including specificity and affinity. The diversity of CDR-H3 loops, arising from their unique biological processes, presents challenges in individually evaluating all loop structures and interactions during extensive screenings [24]. To address these challenges, various DL methods have emerged.

**Figure 8:**
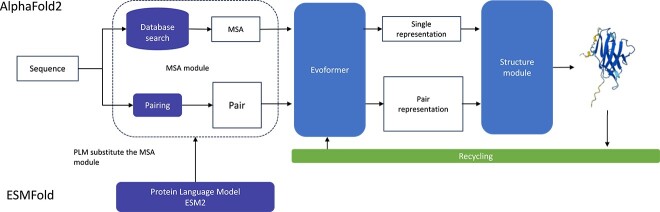
Comparison of the architecture of AlphaFold2 and ESMFold. The Evoformer contains paired attention, outer product difference and triangular update. The Structure modules are based on an IPA module for predicting backbone updates and atom positions. ESMFold uses protein language models (ESM2) to replace the Database-search module.

**Advancement in Protein Folding** A groundbreaking advancement in protein structure prediction was made with AlphaFold2 [106] (Figure 8) and RosettaFold [99], which represent the first two DL-based models that predict protein folding with high accuracy. In particular, AlphaFold2 achieved outstanding results during the 14th Critical Assessment of Protein Structure Prediction (CASP14) (https://predictioncenter.org/casp14/zscores_final.cgi). These models use a multiple sequence alignment (MSA) of homologous proteins as input to trace evolutionary relationships among corresponding residues in genetically related sequences. The algorithm consists of distinct modules: the initial module captures sequence-structure patterns (Evoformer), followed by a module that transforms these patterns into explicit 3D structures and concludes with a physics-based refinement module (Structure module). The successful outcome achieved by AlphaFold2 and RosettaFold implies that a structural representation of the target antigen is typically accessible [107]. Moreover, building on AlphaFold2, AlphaFold-Multimer (AF-Multimer) [108] was created as an end-to-end protein complex structure prediction method. However, MSAs are unsuitable for antibody folding because the CDR H3 loop sequences lack evolutionary data, given their high sequence variability. This raises concerns about the availability and reliability of MSAs in CDR regions [22]. AlphaFold3 [109] introduces a diffusion-based framework for forecasting intricate structures such as proteins, nucleic acids and small molecules. It improves the accuracy of Ab–Ag prediction in contrast to AlphaFold-Multimer. The Evoformer and Structural module have been replaced with simplified MSA processing and a PairFormer block, respectively, reducing prediction time.

**Figure 9:**
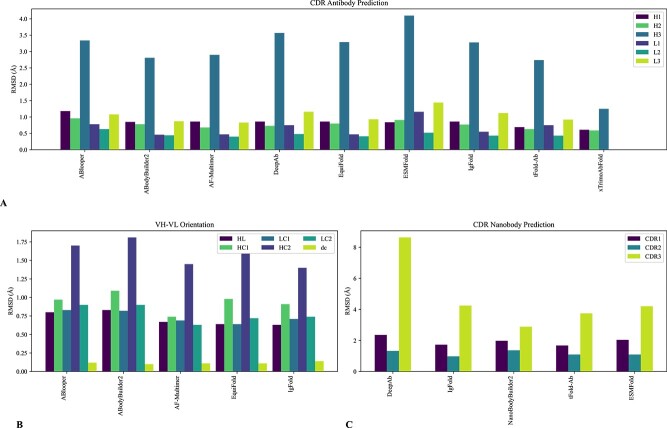
(A) Comparison of the method in predicting the CDR regions of standard antibodies in terms of RMSD. (B) Average absolute error in the five angles (Hl, HC1, HC2, LC1, LC2) and distance (dc) that fully characterize VH-VL orientation, as described in Abanades *et al*. [110]. All methods demonstrate accuracy in predicting the angles and the dc vector. However, small deviations in these angles can significantly affect the binding site structure. (C) Comparison of the RMSD of the CDR regions of nanobodies. The data are sourced from Wu *et al*. [111], except for ABodyBuilder2. The H3 loop (CDRH3 for antibodies and CDR3 for nanobodies) has a higher distance between the ground truth structure and the predicted one. Showing the difficulty of folding this particular loop. For a qualitative analysis of strengths, limitations and application of the methods, refer to Table 5.

**Advancement in Antibody Folding** Specialized DL approaches tailored for antibody structure prediction demonstrate higher accuracy in predicting CDR loops than models trained for general structure prediction. These methods leverage domain-specific knowledge in antibody structures and significantly improve computational speed, enabling the rapid generation of a large volume of antibody structures. The performance of the methods for Ab-folding are shown in Figure 9. Table 5 summarizes each method’s strengths, limitations and applications.

**Table 5:** DL models for predicting antibody structure. For a quantitative result, refer to Figure 9.

**Name**	**Class**	**Model**	Training Dataset	**Time ($\sim $)**	**Description**	**Ref**
ABlooper	Antibody	E(n)-EGNNs	SAbDab (3.4k struct.)	seconds	**Strengths**: Does not rely on external tools and on MSA (Fast). **Limitations**: Can produce unphysical predictions. **Applications**: CDR loop structure prediction.	[112]
ABodyBuilder2 NanoBody- Builder2	Antibody Nanobody	AF-Multimer	SAbDab (3.8 Ab struct. 1k nano struct.)	seconds	**Strengths**: Reduces problems with physical constraints with OpenMM [120] . **Limitations**: Predicting the structure of CDR H3 and generating physically plausible structures continues to pose a challenge. **Applications**: Fv structure prediction, nanobody structure prediction.	[110]
AF-Multimer	Protein	AF2	PDB	hours	**Strengths**: AF extended to multiple chains, with native support for multi-chain featurization and symmetry handling. **Limitations**: Slow, performance declines rapidly for proteins with over two chains, and has high dimension. **Applications**: Protein-protein complexes.	[108]
DeepAb	Antibody	LSTM + residual NN	OAS (118k seq.) SAbDab (1.7k struct.)	minutes	**Strengths**: Can be used to suggest or identify point mutations. **Limitations**: Relies on Rosetta (slow). **Applications**: Fv structure prediction.	[115]
EquiFold	Protein Antibody	SE(3)- equivariant NN	SAbDab (6.8k struct.)	seconds	**Strengths**: Do not rely on MSA or pLM, having fewer parameters (faster). **Limitations**: Can produce unphysical predictions. **Applications**: Design of mini-proteins and Fv structure prediction.	[117]
ESMFold	Protein	ESM2	PDB (325k struct.) AF2 augmentation (12M struct.)	hours	**Strengths**: Folds using only the sequence information without relying on MSA. **Limitations**: High model dimensionality. **Applications**: Protein structure prediction.	[84]
IgFold	Antibody	AntiBERTy + Graph transformer+ IPA	SAbDab (4.2k structures) OAS (folded with AF2) (38.2k struct.)	minutes	**Strengths**: Uses AntiBERTy embeddings and reduces problems with physical constraints. **Limitations**: Use of Rosetta and produces just backbone structures [121] for final refinement. **Applications**: Fv and nonobody structure prediction.	[114]
tFold-Ab	Antibody	AF-Multimer	SAbDab (9.5k struct.)	seconds	**Strengths**: Use of PLM with AF-Multimer. **Limitations**: Choise of the PLM is not well studied. **Applications**: Fv and nanobody structure prediction.	[111]
xTrimoABFold	Antibody	AF-Multimer	PDB (18.9k struct.)	seconds	**Strengths**: Use of AntiBERTy embeddings and fast template search algorithms. **Limitations**: Does not considers complex to further improve the prediction. **Applications**: Fv and nanobody structure prediction.	[118]

For instance, ABlooper [112] employs an E(n)-Equivariant Graph Neural Networks (E(n)-EGNNs [113]) that directly operates on 3D coordinate data from structure files to predict the positions of backbone atoms for the six CDR loops. IgFold [114], an extension of AntiBERty, offers improved average predictions than ABlooper and DeepAb, particularly for nanobodies, using template structures. DeepAb [115] is a bidirectional Long Short-Term Memory (LSTM) pre-trained on 100k paired sequences from the OAS database. It separates sequence embeddings into structural clusters, used for structural predictions with Rosetta [116]. However, its dependence on Rosetta slows down the method compared with IgFold and ABlooper, as shown in Table 5 [24, 114].

The following folding methods were developed to overcome the challenge of MSA. ESMFold leverages ESM-2, which provides a comprehensive embedded representation of protein sequences and serves as a valuable alternative to MSAs [84] (refer to Figure 8 for more detail). EquiFold [117] uses a coarse-grained structure representation model, eliminating the need for MSA or pLM. This speeds up predictions for a given target sequence. EquiFold and AF-Multimer show promise in antibody structure prediction. Significant progress has been made with ABodyBuilder2 [110], which consists of four independently trained DL models to predict an ensemble of antibody structures. These models represent an antibody-specific adaptation of AF-Multimer’s structure module. ABodyBuilder2 forecasts CDR-H3 loops with a Root Mean Square Distance (RMSD) of 2.81 Å, showcasing an improvement over AF-Multimer, while achieving significantly faster computational speeds. AbodyBuilder2 is part of the ImmuneBuilder model ensemble, along with NanoBodyBuilder2 for nanobodies and TCRBuilder2 for T-Cell receptors. NanoBodyBuilder2 predicts CDR-H3 loops with an average RMSD of 2.89Å. Other models that use an AF-Multimer-based architecture as ABodyBuilder2 are tFold-Ab [111], and xTrimoABFold [118]. In general, the error level of these methods can be considered to be in the same order as the X-ray resolution, which has a mean value of 2 Å in the PDB (https://www.rcsb.org/stats/distribution-resolution). This can be regarded as an interesting result [119].

### Antibody–antigen interaction prediction as a key element in effective antibody design

**Figure 10:**
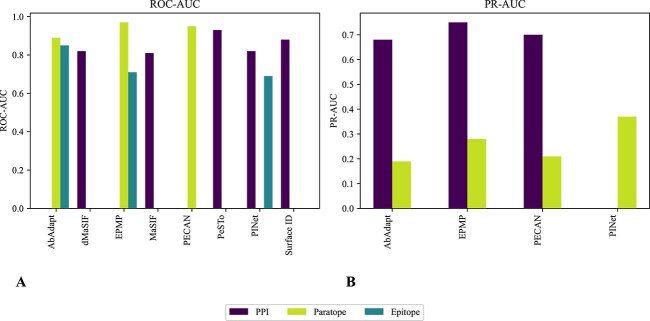
The methods for antibody–antigen interactions are compared based on the Area Under the ROC Curve (ROC-AUC) (A), and the Area Under the Precision-Recall Curve (PR-AUC) (B). As mentioned in the text, epitope prediction yields lower values compared with paratope prediction, since epitopes can be found on the entire pathogen surface, whereas paratopes are found only in VH and VL. To compare these methods in terms of strengths, limitation and application, refer to Table 6.

Once the structures of the antibody and antigen are available, they become valuable assets for assessing their binding potential (Figure 4). One of the initial steps in Ab design often involves accurately predicting the paratope and/or epitope regions. While Ab–Ag interactions are technically a subset of protein–protein interactions (PPIs), it is clear that these interactions and their interfaces have unique characteristics. These distinctive properties make general protein interaction prediction methods less suitable for antibody-related applications [23]. Refer to Figure 10 and Table 6 for an overview of the methods that will be presented in the following subsections.

**Table 6:** Overview of the antibody–antigen interactions methods (complex = complx.). For a comparison of the methods in term of AUC-ROC and AUC-PR, refer to Figure 10.

**Name**	**Class**	**Model**	Training dataset	**Description**	**Ref**	
AbAdapt	Antibody	DNN	PDB (722 Ab-Ag complx.)	**Strengths**: Takes sequences as an input. **Limitations**: Lower performances compared with the other methods; did not perform well in quality of top-scoring poses and speed. **Applications**: Ab-Ag modeling and docking.	[128]
dMaSIF	Protein	GCNN	PDB (4.6k protein complx.)	**Strengths**: Generating molecular surfaces on the fly (fast). **Limitations**: Geometrical features do not boost performances. **Applications**: Interface site prediction and ultra-fast PPI search.	[129]
EPMP	Antibody	**Paratope**: CNN + GNN **Epitope**: GCN + GAT	**Paratope**: AbDb (308 Ab) **Epitope**: SabDab + ZDock [130] (142 Ag) [131, 132, 133]	**Strengths**: Distinct and asymmetric architecture for paratope and epitope. **Limitations**: Does not consider chemical features. **Applications**: Paratope-epitope prediction.	[126] [134]
MaSIF (-Search)	Protein	GCNN	PRISM [135] PDBbind [132, 133] SAbDab ZDock [130] (3k protein complx.)	**Strengths**: Pioneer of the fingerprint method. **Limitations**: Use of pre-computed libraries (slow). **Applications**: Pocket classification, interface site prediction, Ultra-fast PPI search, Protein design.	[136]
PECAN	Antibody	GCN	**Paratope**: [137] (205 Ab) **Epitope**: EpiPred [138] DBD5 [130] (118 Ag)	**Strengths**: Attention layer explicitly encodes the context of the partner. **Limitations**: Use a symmetric architecture for Ab and Ag not considering the differences. **Applications**: Paratope-epitope prediction.	[125]
PeSTo	Protein	Transformer	PDB (376k protein-protein protein-non protein complex.	**Strengths**: Can predict nucleic acids, lipids, ions, and small molecules interfaces. **Limitations**: Slight decrease in performance for non-protein structures. **Applications**: Protein bind interface prediction.	[124]
PINet	Antibody	GDNN	DBD5 (189 protein complx.) DBD3 (60 protein cmplx.) MaSIF (2.7k protein complx.) EpiPred (118 Ab-Ag complx.)	**Strengths**: PPI as a segmentation task. **Limitations**: Need for a convolutional layer to incorporate biophysical properties better. **Applications**: PPI and Ab-Ag interactions.	[127]
Surface ID	Antibody	GCNN	SAbDab (2.7k Ab-Ag complx.)	**Strengths**: Algorithm for high-throughput surface comparison. **Limitations**: The use of MaSIF slows down the method. **Applications**: PPI classification, epitope - paratope clustering, antibody discovery.	[139]

#### GNN-based methods

Protein structures are represented as graphs to analyze protein–ligand interactions (PLI). Several GNN-based methods have been developed in existing literature, particularly using geometric deep learning (GDL). GDL [122, 123] encodes the geometric understanding of data into DL models, enabling the capture of spatial relationships and connectivity in non-Euclidean domains, such as graph and manifold data.

An example of interface interaction, not just limited to PPI, is PeSTo. PeSTo [124] is a groundbreaking parameter-free geometric transformer that directly operates on the atomic components of a protein structure. This approach can accurately predict specific regions on a protein surface that have the potential to interact with other proteins, nucleic acids, lipids, ions and small molecules.

In the context of Ab–Ag interactions, more specialized methods have emerged. For example, PECAN [125] employs a ‘symmetrical’ GCN for predicting both paratopes and epitopes. This model incorporates information for both components within a unified framework during training, considering both antibody and antigen structures. PECAN achieves a PR-AUC of 0.70 for paratope prediction and 0.21 for epitope prediction. Paratope prediction achieves better results than epitope prediction because its location on the antibody structure is known, whereas epitope prediction is challenging as it can be located at any region of the pathogen’s surface. In contrast, EPMP [126] proposes a novel approach for epitope and paratope prediction, considering that the epitope’s position depends on both antibody and antigen structures, while the paratope’s position is independent of the antigen. Based on this, EPMP develops separate prediction models, Para-EPMP and Epi-EPMP, using a combination of sequence and structural graphs for paratope prediction and relying on structural information for epitope prediction. This framework achieves a PR-AUC of 0.75 for paratope prediction and 0.28 for epitope prediction. Finally, PINet [127] uses a geometric deep neural network that is acutely aware of antibodies and antigens, achieving an impressive PR-AUC score of 0.45 for paratope–epitope prediction and 0.37 for epitope prediction, demonstrating state-of-the-art performance in epitope prediction.

**Fingerprint-based methods** MaSIF [136] pioneers the use of GDL for predicting PPIs. MaSIF generates protein fingerprints by breaking down protein surfaces into patches, considering various properties such as amino acid sequence, structural elements and functional motifs. These fingerprints are processed using a Geometric Convolutional Neural Network (GCNN) to identify patterns and relationships among patches, aiding in the identification of ligand binding and protein interactions. The authors used MaSIF to perform different tasks, such as pocket classification (MaSIF-ligand, ROC-AUC: 0.95), interface site prediction (MaSIF-site, ROC-AUC: 0.81) and ultra-fast PPI search (MaSIF-search, ROC-AUC: 0.99). In this work [140], the author developed MaSIF-seed to design new proteins with the concept of the fingerprint. Despite its versatility, MaSIF’s reliance on pre-computed features and meshes results in slow, memory-intensive computations. dMaSIF [129] enhances protein structure analysis by directly operating on raw 3D coordinates and atom types, efficiently generating molecular surfaces on the fly, and overcoming the limitations of MaSIF. It introduces a novel geometric convolutional layer, resulting in faster and more memory-efficient performance than MaSIF, achieving similar outcomes—ROC-AUC of 0.87 vs. 0.85, (MaSIF-site) in site identification, and 0.82 vs. 0.81 (MaSIF-search) in identifying binding partners—but completing tasks 600 times faster.

Focusing specifically on Ab–Ag interactions, Surface ID [139], based on MaSIF, utilizes the concept of fingerprinting for rapid surface comparison. It includes a distinct grouping and alignment algorithm for protein clustering based on function, which helps with visualization and supports *in silico* screening for potential binding partners. Despite its interesting results in epitope and paratope clustering and *de novo* antibody discovery, Surface ID is hindered by its slow speed due to reliance on MaSIF for surface patch generation and a lack of structural flexibility crucial for studying Ab–Ag interactions.

#### Sequence-based methods

An alternative approach for Ab–Ag interactions is to employ sequences rather than structures. For instance, AbAdapt [128], a web server that takes antibody and antigen sequences as input, models their 3D structures (Repertoire Builder [141]), predicts epitopes and paratopes (deep neural networks, DNN) and performs docking using existing tools for local (Hex [142]) and global docking (PIPER [143]). The method achieved a PR-AUC of 0.683 for paratope prediction and 0.194 for epitope prediction. The decrease in performance compared with the structural-based method may be due to the deeper understanding of 3D interactions offered by structural data compared with sequences.

The methods discussed here overlook conformational flexibility, crucial for proteins to assume various 3D structures during interactions. To better represent binding flexibility in Ab–Ag interactions, it is essential to integrate conformational flexibility into Ab–Ag complex modeling. This can be achieved, for example, through folding predictions [144].

### Docking as an essential component of antibody design and testing

Accurate paratope–epitope prediction is important to narrow down the search space for docking [19]. Docking is a process that predicts the binding mode and relative positions of protein–ligand complexes. Molecular docking consists of two essential stages: sampling, which involves generating diverse conformations of a rigid 3D ligand to explore its conformational space, and scoring, assessing the binding affinity of each protein–ligand complex (pose). Although typically viewed independently, these stages can be interconnected, with scoring functions influencing the sampling process. Protein docking methods are broadly categorized into flexible and rigid body, which is faster and less accurate than flexible docking.

**Figure 11:**
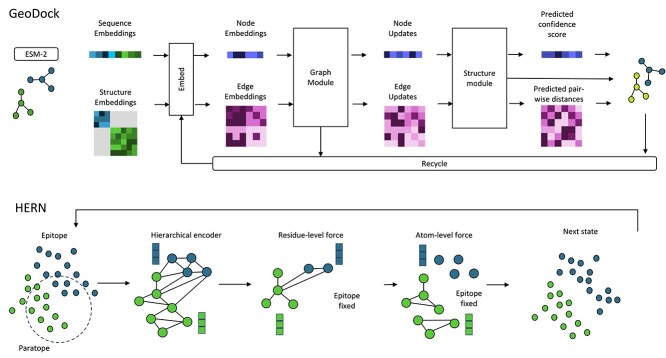
In this figure, two different methods of performing Ab–Ag docking are presented. First, we have the Geodock model, which has a similar architecture to AlphaFold2 (refer to Figure 8) for docking two proteins. Geodock relies on sequence and structural embeddings and uses a transformer to consider both local and global information. Specifically, the Graph modules contain paired attention, outer product difference and triangular update. The Structure modules are based on an IPA module for predicting backbone updates. Another similar architecture, DockGPT, is employed for antibody design. On the other hand, alternative approaches use GNNs. For example, HERN employs a hierarchical message-passing network for paratope design using GNNs. For more details on GeoDock and HERN, refer to Table 7.

Docking is widely used to assist different tasks in drug design [158–160]. For instance, it plays a crucial role in optimizing molecular interactions to enhance drug efficacy. The method presented in the following sections about docking are compared in Table 7 and some of them are represented in Figure 11. An interesting example of flexible protein–ligand docking is GeoDock [151], which employs an AF-based architecture (graph and structure modules). It excels at accommodating conformational changes in both proteins and ligands, making it versatile for studying various protein–ligand interactions. GeoDock’s innovation lies in its ability to handle flexible ligands by encoding their flexibility into molecular graphs. Additionally, incorporating attention mechanisms into MolGCNs allows the model to focus on the most relevant parts of molecular graphs for accurate prediction of binding affinities. This method can be an interesting starting point for Ab–Ag docking.

**Table 7:** Methods for antibody-antigen docking. The dataset part shows the number of protein or Ab–Ag complexes (complx). To compare designs, we consider AAR and RMSD between the original and generated sequences and structures. For docking evaluation, we utilize DockQ[157] and success rate (SSR) to compare the original docked complex with the predicted one. In this table, Transformers are abbreviated as TF. ^*^BC40 is available at https://drug.ai.tencent.com/protein/bc40/download.html.

**Name**	**Class**	**Model**	Training dataset	**Design**	**Docking**	**Description**	**Ref**
DLAB	Antibody	CNN	SAbDab (1.2k Ab–Ag complx.)	-	-	**Strengths**: Improved pose-ranking. **Limitations**: Use of rigid docking instead of flexible docking. **Applications**: Early-stage virtual screening of Ab (known Ag).	[145]
DockGPT	Antibody	TF	BC40^*^ 37k chains DIPS [146] (33k complx.) SAbDab (2.4k Ab–Ag complx.)	RMSD H1: 1.11 Å H2: 1.02 Å H3: 1.88 Å	DockQ: 26.1%	**Strengths**: Circumvents explicit training on bound structures and offers a natural approach to modeling conformational flexibility in complex prediction. **Limitations**: use only single atom type and threshold to provide the model with interface and contact information. **Applications**: Flexible and site-specific protein docking; Dock and CDR design for a specific epitope.	[147]
dyMEAN	Antibody	MEAN	SAbDab Design: 3k Ab Docking: 60 Ab–Ag complx.	AAR Full: 74.96% CDRs: 60.07% H3: 43.65%	DockQ Full: 41.2% CDRs: 39.6% H3: 40.9%	**Strengths**: Multi-channel encoder addresses the issue of varying numbers of atoms in different residues in full-atom modeling. **Limitations**: Cannot design rational antibodies [148]. **Applications**: CDR design and docking considering the epitope structure and Ab incomplete sequence.	[149]
GeoDock	Protein	TF	DIPS [146] (36k complx.) DB5.5 [150] (178 complx.)	-	SSR: 41%	**Strengths**: Uses sequence and structure embeddings. **Limitations**: Does not outperforms methods that use sampling and re-ranking. **Applications**: Flexible protein-protein docking.	[151]
HERN	Antibody	GNN	SAbDab (3k Ab–Ag complx.)	H3 AAR: 34.1%	Paratope DockQ: 43.8 % SSR: 100%	**Strengths**: Represents binding interface as a dynamic hierarchical graph. **Limitations**: Needs to be combined with epitope prediction approaches, focus only on CDR-H3. **Applications**: Paratope docking and design given the epitope.	[152]
Peng *et al*.	Antibody	AbDesign: MC-EGNN AbDock: IPA	SAbDab	RMSD: 2.56 Å AAR: 36.47%	DockQ H chain: 26% CDRH: 30% H3: 44%	**Strengths**: Integrates generative diffusion models for diverse candidate sampling. **Limitations**: Depends on the presence of Ab–Ag complex structures for optimization. **Applications**: CDR design and docking to improve binding affinity.	[153]
PointDE	Protein Antibody	PMLP	DOCK- GROUND [154] (61 complx.) IEDB [155] (659 Ab–Ag complx.)	-	SSR proteins: 65.6% Ab–Ag: 56.6%	**Strengths**: First to use point cloud for protein docking evaluation. **Limitations**: Uses just PDB information. **Applications**: Docking evaluation.	[156]

Focusing more on a tailored method, DLAB [145] improved docking pose ranking and identified antibody–antigen pairs with higher accuracy potential by retraining a CNN with a dataset of 759 antibody–antigen complexes.

To assess protein docking, a novel tool called PointDE was introduced in a study by Chen *et al*. [156]. PointDE employs multiple PointMLP (PMLP) [161] applied on 3D point cloud data to assess the quality of protein docking by evaluating whether a docking decoy closely resembles the native structure. This method was also applied to evaluate antibody–antigen complexes.

Molecular docking faces challenges in accurately representing binding, particularly with flexible molecules like antibodies and their protein counterparts, and the accuracy of protein docking is constrained by algorithmic limitations and structural uncertainties, particularly in the CDR-H3 loop [162–164].

#### Jointly docking and design antibody–antigen complexes

As mentioned in [164], the main component of modeling antibody–antigen complexes, including structure prediction, paratope–epitope prediction and docking (as shown in Figure 4), could potentially be simultaneously achieved in a single step using a generative modeling approach.

The Sculptor [165] method, which utilizes a variational autoencoder generative model, explores the conformational space of a single fold. While Sculptor combines generative modeling with docking and loop dynamics for epitope-specific design, DockGPT [166] uses an encoder–decoder module with triangle multiplication and pair-based attention to perform *de novo* CDR loop design using fine-tuned antibody–antigen complex encoding. HERN [152] (see Figure 11) uses a hierarchical message-passing network for docking and designing paratopes. It predicts atomic forces to refine binding complexes during docking iteratively. Its autoregressive decoder progressively docks paratopes and assists in selecting residues based on the interface geometry for the paratope design. dyMEAN [149], which outperformed HERN, offers an end-to-end solution using Multi-channel Equivariant Attention Network (MEAN), where only the epitope and the incomplete 1D sequence are known. The latter is updated iteratively using adaptive multi-channel message passing, enabling it to process protein residues of various sizes. The process concludes by docking the refined antibody to the epitope based on the shadow paratope (a cloned representation of the paratope surrounding the epitope) alignment. Peng *et al*. [153] developed a diffusion-based antibody optimization pipeline to enhance binding affinity. The pipeline consists of two main stages: AbDesign for generating antibody sequences and structures, and AbDock, a docking model for screening designed CDRs. The model is based on Invariant Point Attention (IPA) for modeling antibody–antigen complexes and utilizes generative diffusion models (Multi-Channel Equivariant Graph Neural Network, MC-EGNN) to sample diverse candidates. Notably, the AbDock method demonstrated exceptional capabilities, achieving outstanding results in various evaluation metrics. For instance, outperformed HERN in H3 design and docking (DockQ 44% vs. 43% vs. 37% (HERN relaxed)).

### Enhancing antibody binding affinity through *in silico* affinity maturation

**Figure 12:**
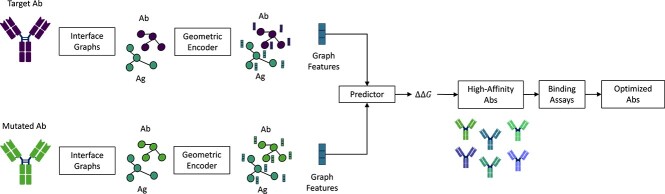
Approximate workflow of the presented methods. The antibodies, including the mutated variant, undergo testing using *in silico* methods (geometric encoders, e.g. GNNs and Transformers) to identify high-affinity mAbs. Subsequently, these identified antibodies are evaluated *in vitro*. The input graph can be considered for atoms or residues.

While structural modeling, paratope–epitope prediction and docking methods serve as the foundation for identifying potential binding molecules through virtual screening, antibodies initially identified using these methods often exhibit weak binding [145]. *In vitro* affinity maturation methods (e.g. random mutagenesis) have demonstrated effectiveness in enhancing antibody binding to target proteins. However, these approaches are both time-consuming and labor-intensive [167]. Recent advancements have introduced *in silico* affinity maturation techniques to address this limitation. These methods use machine learning to predict and identify mutations improving binding affinity (see Figure 12).

GeoPPI [171] involves two components: first, training a GAT on topology features from protein structures using self-supervised learning, and second, training a gradient-boosting tree (GBT) on features derived from both wild-type and mutant counterpart. The combined model predicts $\Delta \Delta $G values when amino acids are replaced.

To tackle the challenge of enhancing antibodies for broader neutralization against SARS-CoV-2 variants, Shan *et al*. [73] introduced a Transformer-based architecture, as previously discussed in the subsection on antibody design using structures. This network enhances antibodies effectively, emphasizing the need for broad neutralizing activity across diverse variants. The model identifies crucial residue pairs near the protein interface influencing binding affinity and predicts mutation effects on protein complexes by comparing wild-type and mutated embeddings. Demonstrating a moderate to high correlation with experimental binding data, it surpasses GeoPPI [171] and other recent methods for predicting single mutation effects. However, it is important to note that this method is specifically designed for SARS-CoV-2 variants.

Another inspiring work GearBind [168], a pre-trainable deep neural network for *in silico* affinity maturation, effectively extracts geometric representations from wild-type and mutant structures, predicting binding free energy change ($\Delta \Delta $$G_{bind}$). Using an ensemble model based on self-supervised pre-trained GearBind, the authors successfully optimize the affinity of CR3022 to the spike (S) protein of the SARS-CoV-2 Omicron strain, achieving a high success rate with up to a 17-fold affinity increase. Moreover, GearBind outperformed the method presented in [73] (RMSE: 1.403 vs 1.539 and PearsonR: 0.62 vs. 0.58). Results are in Table 8.

**Table 8:** Method for antibody affinity maturation compared in terms of Pearson correlation coefficient (PearsonR) and Root Mean Square Error (RMSE). The methods are compared for single mutation (SM) and multiple mutation (MM) using S1131 [172] and M1707 [173] respectively, with the exception of GearBind. The ’Application’ is not shown in the ’Description’ column as all methods predict mutational effects on binding affinity. (structures = struct.)

**Name**	**Class**	**Model**	Training dataset	**PearsonR**	**RMSE**	**Description**	**Ref**
GearBind	Antibody	Geometric GNN	SKEMPI v2 (6k mutations) PDB (123k struct.)	SM: 0.62	SM: 1.40 Å	**Strengths**: Use of contrastive learning to detect destabilizing mutations. **Limitations**: Mutant structure generation time should be improved.	[168]
GeoPPI	Antibody	GAT	PDB-BIND [169] 3DComplexes [170] (13k mutations)	SM: 0.58 MM: 0.74	SM: 2.01 Å MM: 2.21 Å	**Strengths**: Self-supervised learning to reconstruct the coordinates of the perturbed side chains. **Limitations**: Lower performance for single mutation compared with the other two presented methods.	[171]
Shan *et al*.	Antibody	Transformer	SKEMPI V2.0 (5k mutations)	SM: 0.65 MM: 0.59	SM: - MM: -	**Strengths**: The attention network learns to identify key residue pairs near the protein interface that contribute to binding affinity. **Limitations**: Operates at the residue level and does not consider the atom level.	[73]

### Computational methods for assessing developability as a final check for your *in silico* models

Assessing developability is vital for evaluating monoclonal antibody (mAb) candidates with minimal risks. Key aspects include stability, aggregation, immunogenicity and chemical degradation [24, 174]. This evaluation should use both *in vitro* and *in silico* methods. DL generates diverse antibodies quickly. However, *in vitro* testing is necessary to validate their ability to bind to the target antigen and detect any developability issues. This process requires significant resources. Thus, preliminary screening to identify low-risk sequences or structures is essential. While this survey lacks detailed procedures due to the broad nature of the topic, Khetan *et al*.’s review [174] offers a comprehensive overview of databases, tools and guidelines for developability assessment.

## Conclusion

In the field of antibody engineering, integrating artificial intelligence, specifically DL, with traditional methods shows promise in developing therapeutic drugs. While the accurate prediction of antibody and antigen structures has resulted in significant advancements, demonstrating the importance of DL in antibody development, there are still challenges to overcome. One crucial challenge is refining the prediction of paratope–epitope interactions, which is essential for improving the docking process. Additionally, accurately representing antibody–antigen docking is complex due to algorithmic limitations and uncertainties surrounding the structure of the CDR-H3 loop. The emerging approach of simultaneously docking and designing antibodies offers a comprehensive strategy to overcome these obstacles. Furthermore, recognizing the flexibility of antibodies is crucial for understanding antibody–antigen interactions, for example starting from folding methodology. Lastly, the limited availability of data in the literature poses a significant limitation that requires attention to fully harness the potential of DL in biology and medicine. Although antibody design techniques are valuable for augmenting data in various tasks, they often rely on AAR-type metrics, disregarding the possibility that different antibody sequences can bind to the same antigen. As a result, validating these methodologies requires laborious and resource-intensive *in vitro* testing. One potential solution involves identifying design metrics that align with antibody characteristics and exploring DL methodologies to evaluate developability.

In summary, advancements in DL methods show promise in optimizing antibody development workflows and improving the effectiveness and scalability of biotherapeutics.

**Key Points**

- Antibodies are crucial for immune responses and widely used as biotherapeutics.
- Integrating computational methods into traditional techniques is expected to enhance antibody availability and affordability.
- Deep learning advancements offer potential for optimizing antibody development workflows.
- While progress has been made in predicting antibody and antigen structures, challenges remain in precise paratope and epitope prediction, docking and data availability.
- Future research aims to simultaneously dock and design antibodies, considering antibody flexibility in the design process.

## Data Availability

No new data were generated or analysed in support of this research.

## Author Biographies

**Sara Joubbi** is a PhD student in Computer Science at the University of Pisa in collaboration with the Fondazione Toscana Life Sciences. Her research primarily focuses on image analysis and the development of antibodies for infectious diseases using deep learning.

**Alessio Micheli** is full professor at the Department of Computer Science of the University of Pisa, where he is the coordinator of the Computational Intelligence and Machine Learning research group. His research interests include deep learning for complex data, including graphs and networks, with applications to bio/cheminformatics.

**Paolo Milazzo** is associate professor at the Department of Computer Science of the University of Pisa. He coordinates the research group on Biosystems Modelling. His research interests include computational systems biology, bioinformatics and modeling and simulation of complex biological systems.

**Giuseppe Maccari** is senior scientist and coordinator of the Data Science for Health (DaScH) Lab in Fondazione Toscana Life Sciences. His research focuses on computational molecule design for therapeutic and prophylactic medicines, encompassing areas such as antibody and vaccine design.

**Giorgio Ciano** is a researcher at the Data Science for Health Lab (DaScH Lab) of Fondazione Toscana Life Sciences. His main research interests include deep learning and computer vision applied to the biology field.

**Dario Cardamone** is a researcher in Fondazione Toscana Life Sciences and member of the Data Science for Health Lab. His research interests include deep learning with applications in vision, language and biology.

**Duccio Medini** is a scientist and pharmaceutical executive, currently serving as R3 Program Director at Wellcome Leap, a global ARPA for Health, and as Strategic Data Science Director at Fondazione Toscana Life Sciences.
